# Targeted multidomain intervention for complex mTBI: protocol for a multisite randomized controlled trial in military-age civilians

**DOI:** 10.3389/fneur.2023.1085662

**Published:** 2023-06-30

**Authors:** R. J. Elbin, Alicia Trbovich, Melissa N. Womble, Anne Mucha, Sheri Fedor, Katie Stephenson, Cyndi Holland, Christina Dollar, Patrick Sparto, Kori Durfee, Charity G. Patterson, Clair N. Smith, Theodore J. Huppert, David O. Okonkwo, Michael W. Collins, Anthony P. Kontos

**Affiliations:** ^1^Office for Sport Concussion Research, Department of Health, Human Performance and Recreation, University of Arkansas, Fayetteville, AR, United States; ^2^UPMC Sports Medicine Concussion Program, Department of Orthopaedic Surgery, University of Pittsburgh Medical Center, Pittsburgh, PA, United States; ^3^Inova Sports Medicine Concussion Program, Fairfax, VA, United States; ^4^UPMC Centers for Rehab Services, Pittsburgh, PA, United States; ^5^Inova Physical Therapy Center, Fairfax, VA, United States; ^6^College of Osteopathic Medicine, University of New England, Biddeford, ME, United States; ^7^School of Health and Rehabilitation Sciences, Department of Physical Therapy, University of Pittsburgh, Pittsburgh, PA, United States; ^8^Department of Electrical and Computer Engineering, Swanson School of Engineering, University of Pittsburgh, Pittsburgh, PA, United States; ^9^Department of Neurological Surgery, School of Medicine, University of Pittsburgh, Pittsburgh, PA, United States

**Keywords:** mild traumatic brain injury, concussion, clinical profiles, treatment, chronic

## Abstract

**Background:**

Mild traumatic brain injury (mTBI) affects ~18,000 military personnel each year, and although most will recover in 3–4 weeks, many experience persisting symptoms and impairment lasting months or longer. Current standard of care for U.S. military personnel with complex mTBI involves initial (<48 h) prescribed rest, followed by behavioral (e.g., physical activity, sleep regulation, stress reduction, hydration, nutrition), and symptom-guided management. There is growing agreement that mTBI involves different clinical profiles or subtypes that require a comprehensive multidomain evaluation and adjudication process, as well as a targeted approach to treatment. However, there is a lack of research examining the effectiveness of this approach to assessing and treating mTBI. This multisite randomized controlled trial (RCT) will determine the effectiveness of a targeted multidomain (T-MD) intervention (anxiety/mood, cognitive, migraine, ocular, vestibular; and sleep, autonomic) compared to usual care (behavioral management) in military-aged civilians with complex mTBI.

**Methods:**

This study employs a single-blinded, two-group repeated measures design. The RCT will enroll up to 250 military-aged civilians (18-49 yrs) with a diagnosed complex mTBI within 8 days to 6 months of injury from two concussion specialty clinics. The two study arms are a T-MD intervention and a usual care, behavioral management control group. All participants will complete a comprehensive, multidomain clinical evaluation at their first clinical visit. Information gathered from this evaluation will be used to adjudicate mTBI clinical profiles. Participants will then be randomized to either the 4-week T-MD or control arm. The T-MD group will receive targeted interventions that correspond to the patient’s clinical profile (s) and the control group will receive behavioral management strategies. Primary outcomes for this study are changes from enrollment to post-intervention on the Neurobehavioral Symptom Inventory (NSI), Patient Global Impression of Change (PGIC), and functional near-infrared spectroscopy (fNIRS). Time to return to activity (RTA), and healthcare utilization costs will also be assessed.

**Discussion:**

Study findings may inform a more effective approach to treat complex mTBI in military personnel and civilians, reduce morbidity, and accelerate safe return-to-duty/activity.

**Ethics and dissemination:**

The study is approved by the University of Pittsburgh Institutional Review board and registered at clinicaltrials.gov. Dissemination plans include peer-reviewed publications and presentations at professional meetings.

**Clinical Trial Registration:**

www.clinicaltrials.gov, identifier: NCT04549532.

## 1. Introduction

Mild traumatic brain injury (mTBI) affects ~18,000 military personnel each year (1). Although most military personnel with mTBI will recover in 3–4 weeks, many experience symptoms and impairments lasting months or longer, resulting in limited operational capabilities and duty restrictions (2). These “complex” mTBIs may involve vestibular, cognitive, somatosensory, and/or autonomic dysregulation that if left untreated can become chronic in affected personnel (3). This protracted course of recovery adversely affects the health of military personnel, results in productivity losses, and negatively impacts force readiness. In addition, mTBI places a significant burden on the Military Health System. The estimated one-year direct costs for mTBI-related care range up to $30,000 per military personnel (4).

There is growing agreement that mTBI is a heterogeneous injury involving multiple domains of symptoms and impairment (5–7). Research indicates that mTBI may involve different clinical profiles or subtypes that require a comprehensive multidomain evaluation and targeted approach to treatment (8, 9). Each of these clinical profiles is characterized by specific symptoms and impairment that can be targeted with matched treatments. Importantly, complex mTBI typically involves multiple, often overlapping affected domains including but not limited to anxiety/mood, cognitive, migraine/headache, ocular, sleep, vestibular, and autonomic (7, 9). Previous trials have failed to address this heterogeneity in their design, evaluation of clinical outcomes, and application of treatments.

Current standard of care for U.S. military personnel with mTBI involves initial prescribed rest, followed by primary care-based behavioral management (e.g., sleep regulation, stress reduction, hydration, nutrition), and symptom-guided management. Growing evidence suggests that this approach may not be effective for all patients (10–12) and strict rest beyond 48 h may in fact be detrimental to other patients (11, 12). Concurrently, there is evidence that more active and targeted treatment approaches may be beneficial to patients (11, 13, 14). In response to this evidence, researchers and clinicians have begun advocating for more active and targeted treatment approaches for treating the specific clinical profiles or subtypes of symptoms/impairment following mTBI (11, 14, 15).

Previous research on the effectiveness of treatments for mTBI has been limited in that only single treatments [e.g., (16)] have been used and/or multiple treatments have been applied across all patients groups without regard to specific symptoms and impairment or clinical profiles. In addition, when multiple treatments have been applied, separate treatment effects for each intervention were not considered [e.g., (17)]. The T-MD intervention in the current proposal will implement targeted interventions for seven affected domains including: (1) anxiety/mood, (2) cognitive, (3) migraine, (4) ocular, (5) vestibular, (6) sleep, and (7) autonomic. Each intervention will target a specific affected domain (e.g., vestibular rehabilitation for a patient with vestibular impairment and/or symptoms), but not all patients will receive all interventions. This approach better reflects precision clinical care models and may provide a more effective and efficient therapeutic pathway for patients with complex mTBI.

Research on the effectiveness of treatment following mTBI has yet to provide concomitant evidence of changes in the brain that may underly clinical findings. Functional near-infrared spectroscopy (fNIRS) is an optical imaging technique that measures temporal–spatial changes in oxygenated (activation) and deoxygenated (deactivation) hemoglobin concentrations in the cerebral cortex and is highly correlated with cerebral blood flow (CBF). Functional NIRS provides an objective measure of brain injury *via* changes in CBF that correspond to functional impairments in cognition (18) and vestibular function (19). Functional NIRS may provide an objective measure of treatment effectiveness by evaluating changes in brain activation at rest and during a cognitive paradigm in conjunction with clinical outcomes.

The overall objective of this multisite RCT is to determine the effectiveness of a T-MD intervention compared to usual care (behavioral management) in military-aged civilians with complex mTBI (anxiety/mood, cognitive, migraine, ocular, vestibular, sleep, and autonomic domains). The study involves three primary aims: (1) compare using an RCT the effectiveness of T-MD (anxiety/mood, cognitive, migraine, ocular, vestibular, sleep, autonomic domains) intervention to usual care (behavioral management) on improving clinical profile-based outcomes including symptoms, impairments, return to activity (RTA), and healthcare utilization costs in 200 military-aged civilian patients with complex mTBI; (2) to compare changes in brain activation (i.e., CBF) as measured by fNIRS in the T-MD and usual care intervention groups; and (3) to correlate changes in symptoms and impairment with brain activation as measured by fNIRS. We will also explore factors (e.g., demographics, medical history, injury information) that may influence the efficacy of T-MD on NSI, PGIC, and RTA outcomes.

## 2. Methods and analysis

### 2.1. Study design

A prospective, single-blinded, two-group multicenter RCT of T-MD versus usual care (i.e., behavioral regulation) in military-aged civilians with complex mTBI will be conducted at two concussion specialty clinics: (a) University of Pittsburgh Medical Center Sports Concussion Program (Pittsburgh, PA), and (b) Inova Sports Medicine Concussion Program (Fairfax, VA). The current study will employ a two-group repeated measures (baseline, 2-week, 4-week, 3-month) design with permuted block random assignment. The study will be conducted under approvals from University of Pittsburgh Institutional Review Board (IRB) and Human Research Protection Office (HRPO) IRBs. The University of Pittsburgh’s IRB will act as the single site IRB for this project.

### 2.2. Allocation to study arms

Participants will be screened and recruited from two concussion specialty clinics, and randomization will be stratified by site. Within site, a permuted block randomization with random block sizes with a 1:1 allocation ratio will be used. The lead biostatistician will generate the randomization list which will be uploaded into the electronic data capture system. The site coordinator will receive the allocation for each participant once all eligibility information is entered and the participant is deemed eligible for the trial. Participants will be randomized to either the T-MD or usual care intervention arm. Treating clinicians and study research staff will not be blinded to participant group, as they will be employing study procedures and supervising participants in the study. The Principal Investigators will be blinded to participant group assignment throughout the study, including during data analysis.

### 2.3. Participants and eligibility criteria

A total of 250 participants with complex mTBI will be enrolled into the study. We expect complete data for 200/250 (80%) participants per estimated combined attrition rates of 20%. A total of up to 125 participants will be enrolled at each site. Aside from age, the demographics of the sample of participants will not be controlled, as a convenience sample will be enrolled. Based on the combined population demographics of the Pittsburgh and Northern Virginia areas where the trial will occur, we expect participants to reflect closely the racial/ethnic representation from the 2013 Demographics Report of Active-Duty military (20). Specifically, we expect participants to have the following demographic characteristics: White (70%), African American (17%), and other minority (13%). However, given the higher numbers of women treated for concussion at the two sites, we expect the representation of women to be higher in the current study (e.g., 50–60%) compared to the US active-duty military population (e.g., 40%).

Inclusion criteria are age 18–49 years, normal/corrected vision, and diagnosed with a currently symptomatic mTBI with a clear mechanism of injury in the past 8 days-6 months. In addition, participants will have complex mTBI symptoms and/or impairments in at least one of the following domains: anxiety/mood, cognitive, migraine, ocular, vestibular, sleep, autonomic; per a comprehensive assessment, clinical exam/interview, and adjudication process. Exclusion criteria for this study include history of vestibular disorder (e.g., benign paroxysmal positional vertigo, unilateral or bilateral vestibular hypofunction), history of neurological/mental health disorders (e.g., epilepsy, seizure disorders, schizophrenia, bipolar), history of brain surgery/malformations/tumors, diagnosed with cardiac, peripheral or cerebrovascular disease, experienced chest pain or shortness of breath while at rest or with mild exertion, been told by a doctor to only conduct physical activity under medical supervision, previous moderate to severe TBI, <8 days- or > 6 months following current complex mTBI, currently pregnant or become pregnant during study, and/or currently involved in litigation associated with current or previous mTBI, currently on workman’s compensation, previously participated in the study, or previously received clinical concussion care at either participating site within the last 2 years. Participants with a history of mTBI, attention deficit hyperactivity disorder (ADHD), learning disability (LD), migraine, or motion sickness will not be excluded, which will more closely reflect the true characteristics of this population and increase the external validity of the study results.

### 2.4. Measures and instrumentation

#### 2.4.1. mTBI diagnosis criteria

Diagnosis of mTBI will be operationally defined in line with current U.S. military assessments as including the following diagnostic criteria: clear mechanism of injury; Glasgow Coma Scale (GCS) = 13–15; reported or observed signs (e.g., loss of consciousness, amnesia, disorientation/confusion) at time of injury; and/or current reported symptoms (e.g., headache, dizziness, nausea) and/or impairments (e.g., cognitive, balance, visual).

#### 2.4.2. Complex mTBI diagnosis criteria

The complex mTBI diagnosis will be defined as persistent mTBI symptoms that are associated with observable clinical impairments lasting more than a week following injury. The domains and assessments that will be used to screen for complex mTBI are documented in Table 1 below. Participants must demonstrate a positive finding in at least one of the complex mTBI domains to be considered for enrollment into the study. Tests from the NIH Common Data Elements (CDE) (21) set for sport-related concussion will be utilized when available.

**Table 1:** Assessments for determining complex mTBI domains.

Domain	Positive findings/indication for enrollment
Anxiety/Mood	Global Severity Index (GSI) T-score > 50; or CP-Screen Anxiety(A)/Mood(M) Avg score ≥ 2 or 1+ individual item = 3 BSI-18 subscale T score ≥ 63 (Overall, A, M) **Subdomains**: Anxiety (A): 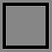 Mood (M): 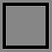 Both (M/A): 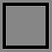
Cognitive	≤7^th^% on one or more ImPACT composite scores; or CP Screen Cognitive Avg score ≥ 2 or 1+ individual item = 3; AND ≤ 30 on ImPACT Impulse Control; AND < 9 on REY 15
Migraine/ Headache	≥2 on ID Migraine- migraine (M); or ≥ Grade 2 (≥ 50) on HIT-6- headache (HA); or CP-Screen Migraine Avg score ≥ 2 or 1+ individual item = 3 **Subdomains**: Headache (HA) Only: 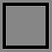 Both (M/HA): 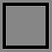 Migraine (M) with ocular/light sensitivity: 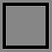 Migraine (M) with noise sensitivity: 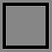 Migraine (M) with motion sensitivity: 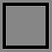
Ocular	≥2 on smooth pursuits or hor/ver saccades, or near point convergence (NPC) ≥10 cm; or CP-Screen Ocular Avg score ≥ 2 or 1+ individual item = 3
Vestibular	≥2 on vestibulo-ocular reflex (VOR) and/or visual motion sensitivity (VMS); or > 9 total errors on mBESS; or CP-Screen Vestibular Avg score ≥ 2 or 1+ individual item = 3
Sleep	≥5 on PSQI global score, or CP-Screen Sleep Avg score ≥ 2 or 1+ individual item = 3
Autonomic	Increase of 3+ (from resting value) on VAS; or Unable to complete BCBT per patient or clinician

Clinical Profiles Screen (CP-Screen), Brief Symptom Inventory-18 (BSI-18), Immediate Post-Concussion Assessment and Cognitive Testing (ImPACT), REY15-Item Memorization Test (REY 15), Headache Impact Test-6 (HIT-6), Pittsburgh Sleep Quality Index (PSQI), Visual Analogue Scale (VAS), Buffalo Concussion Bike Test (BCBT).

#### 2.4.3. Primary and secondary study outcomes

Primary outcomes are the Neurobehavioral Symptom Inventory (NSI), Patient Global Impression of Change (PGIC), and Functional Near-infrared Spectroscopy (fNIRS). These primary outcomes represent different domains of function that are commonly affected following mTBI and provide different sources of information about the injury that inform clinical care decisions (i.e., treatment and/or rehabilitation). The NSI gathers specific information about mTBI symptoms. This measure is comprised of 22 items scored from 0 to 4 (0 total score represents no mTBI-related symptoms, 88 total score represents very severe levels of all 22 mTBI-related symptoms). The PGIC captures patient self-reported assessment of changes in their overall quality of life throughout their mTBI recovery. The prompt for the PGIC is: “Since beginning treatment at this facility, how would you describe the change (if any) in activity limitations, symptoms, emotions and overall quality of life related to your post-concussive condition?” The patient responds on a scale ranging from 0 (No change or condition has gotten worse), to 7 (A great deal better and a considerable improvement that has made all the difference). The fNIRS is a non-invasive imaging tool that measures changes in cerebral blood flow (CBF) and oxygenation in response to a behavioral task. The current study utilizes three neurocognitive tasks (WAIS symbol search and coding, and a flanker task) and a resting condition. Brain activation and deactivation in brain regions of interest (ROIs) will be examined and compared between groups within the first week following enrollment and at approximately four-weeks after enrollment. Secondary outcomes will include data from symptom, neurocognitive, balance, vestibular, sleep, and physical activity assessments (see Table 2). In addition, participant demographics and medical history data will be gathered as part of the normal clinical interview for mTBI (e.g., age, sex, history of mTBI, migraine). A daily text message-based assessment will be used to evaluate each participant’s compliance with their assigned intervention; and to assess frequency and intensity of at-home exercises in between the initial, 2-, and 4-week in-person study intervals. A FitBit Inspire 2 activity monitor will be provided to each participant to track activity and sleep throughout the 4-week intervention period. A detailed description of all study measures, scoring, and validity is presented in Supplemental material (see Description of Measures).

**Table 2:** Assessments used for primary and secondary outcomes.

Primary outcomes	Secondary outcomes
Neurobehavioral Symptom Inventory (NSI)	Behavioral Symptom Inventory (BSI-18)
Patient Global Impression of Change (PGIC)	Dizziness Handicap Inventory (DHI)
Functional Near-infrared Spectroscopy (fNIRS)	Vestibular/Ocular Motor Screening (VOMS)
	Modified Balance Error Scoring System (mBESS)
	Functional Gait Assessment (FGA)
	Clinical Profile Screening (CP-Screen)
	Immediate Post-concussion Assessment and Cognitive Testing (ImPACT)
	Pittsburgh Sleep Quality Index (PSQI)
	Headache Impact Test (HIT-6)
	ID Migraine
	Short-form McGill Pain Questionnaire (SF-MPQ)
	International Physical Activity Questionnaire (IPAQ)
	Buffalo Concussion Bike Test (BCBT)

#### 2.4.4. Interventions for adjudicated concussion clinical profiles and usual care groups

Participants assigned to the T-MD Intervention Group will be prescribed one or more of the intervention domains described in the following sections based on the results of the adjudication process. For example, one participant may be prescribed interventions for anxiety/mood, ocular, and vestibular domains, whereas another participant may be prescribed interventions for anxiety/mood, sleep, and autonomic domains. Each prescribed intervention will be tailored to the specific affected domains and sub-domains, based on clinical consensus (5). For example, within vestibular interventions, one participant may be prescribed only dynamic balance interventions, whereas another participant may be prescribed dynamic gait, gaze stability, and dynamic balance interventions. We will track the specifics of each participant’s intervention to evaluate individual intervention effects. T-MD interventions will be prescribed for a 4-week treatment period (See Description of TMD Interventions document presented in Supplemental material). The participants in the usual care intervention group will be prescribed standardized behavioral management strategies related to activity, sleep, hydration, nutrition, and stress (See Description of Behavioral Regulation Strategies document in Supplemental material).

#### 2.4.5. Return to activity/recovery

Return to activity will be medical clearance to resume full activities based on participant being symptom and/or impairment free at rest, and symptom free following standardized exertion protocols per recent consensus (16). We will assess RTA at 4 weeks and 3 months post-intervention.

#### 2.4.6. Healthcare utilization and costs

Throughout the trial we will record the number of costs incurred by the patient during their clinical care for mTBI including numbers and types of providers involved, CPT codes charged, and the number of work/school absences reported by each participant enrolled into the trial. All CPT coding will be entered from data within each participating hospital system. The CPT codes will include any within-system referrals that the clinical team provides as part of their care. Any care that happens outside of the participating healthsystems, which is rare, will be recorded by the research team.

### 2.5. Procedures

Potential participants with a suspected complex mTBI will be identified during their initial clinic evaluation. Treating clinicians, and a member of the clinical research team, will administer the screening measures as part of their standard clinical exam. At the end of the exam, eligible participants will be informed if they qualify for the study and if they demonstrate interest, they will be referred to an on-site research coordinator. If an eligible individual is not interested, the treating clinician will complete their standard of care treatment plan. Interested participants will be enrolled, consented, and scheduled to complete the initial comprehensive clinical outcome assessments (baseline assessment), including primary and secondary outcome measures, within 5–7 days of study enrollment. After randomization, the participant’s treating clinician will describe the study treatment interventions based on the participant’s group assignment. Participants will be provided detailed instructions and demonstrations of all assigned interventions. At this initial timepoint, research staff will onboard participants for daily text message reminders that will enable participants to self-report daily compliance (yes/no) for completion of their assigned interventions for the 4 weeks of study participation. Text message reminders and self-reported compliance will also be distributed to the usual care group regarding the adherence with behavioral regulation activities (e.g., practice good sleep hygiene).

**Figure 1:**
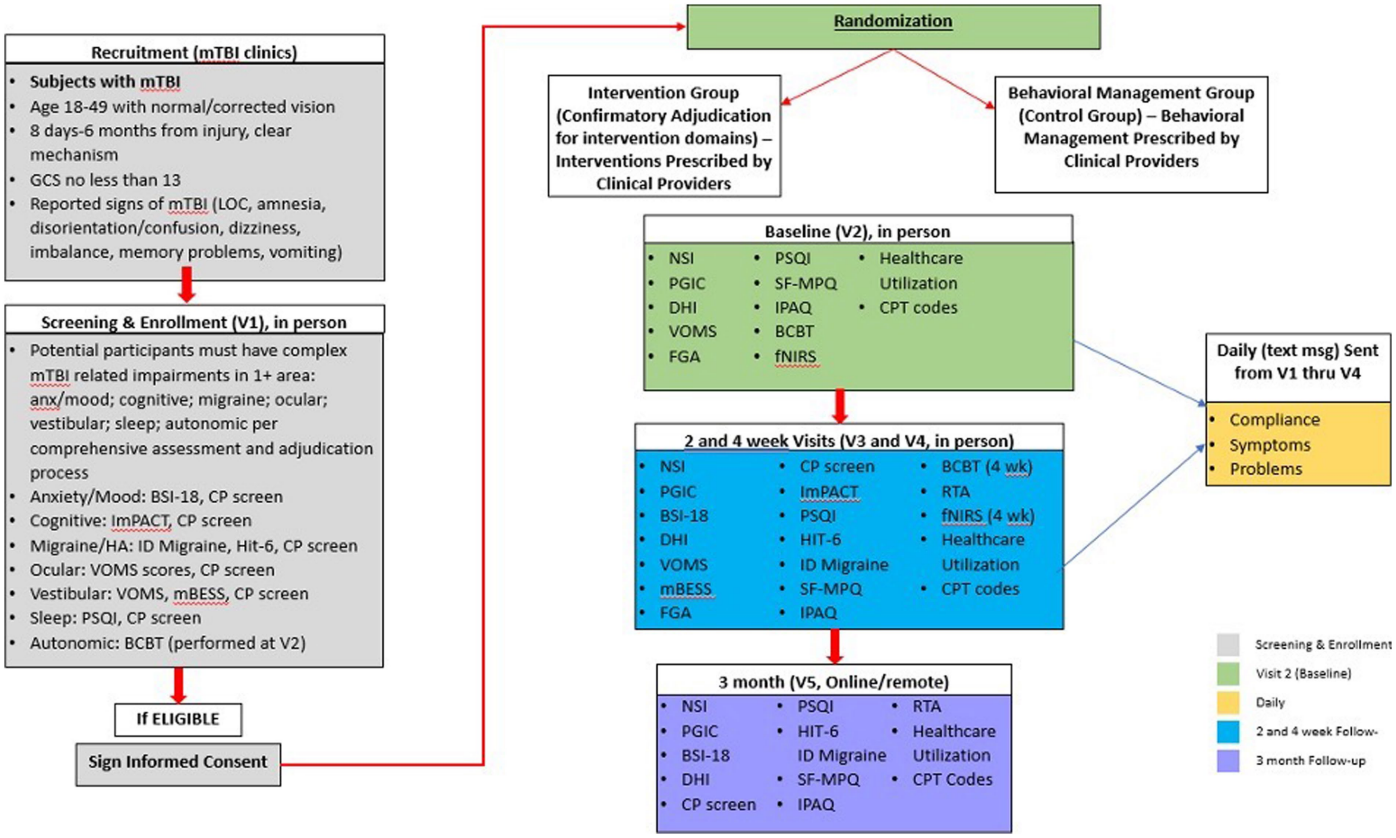
Study procedures for recruitment, screening, randomization, and outcomes assessment timelines for intervention/control groups. Anxiety (Anx), Brief Symptom Inventory-18 (BSI-18), Buffalo Concussion Bike Test (BCBT), Clinical Profiles Screen (CP-Screen), Current Procedural Terminology (CPT), Dizziness Handicap Inventory (DHI), Functional Gait Assessment (FGA), Functional Near Infrared Spectroscopy (fNIRS), Headache (HA), Headache Impact Test-6 (HIT-6), Immediate Post-Concussion Assessment and Cognitive Testing (ImPACT), International Physical Activity Questionnaire (IPAQ), Loss of Consciousness (LOC), Mild Traumatic Brain Injury (mTBI), Modified Balance Error Scoring System (mBESS), Pittsburgh Sleep Quality Index (PSQI), Return to Activity (RTA), REY15-Item Memorization Test (REY 15), Short-Form McGill Pain Questionnaire (SF-MPQ), Vestibular Ocular Motor Screening (VOMS), Visual Analogue Scale (VAS).

After the initial clinical evaluation and randomization into treatment or usual care groups, the clinical research team will adjudicate each participant’s complex mTBI diagnosis. Participant symptoms, impairments, and clinical exam/interview findings will be discussed to substantiate the presence of symptoms/impairments in each of the complex mTBI domains. An adjudication checklist will be completed during this process along with additional information from the clinical exam and interview. At the conclusion of the review and discussion, group consensus will be used to confirm each participant’s complex mTBI profiles and the final clinician-adjudicated and assessment-identified complex mTBI domains will be used to inform the targeted interventions for participants assigned to the T-MD group. At any point during the 4-week intervention of the study, domains can be updated and changed by treating clinicians. The adjudication process is based on the process developed in our TEAM-TBI work (22). A timeline of the study procedures and assessments is summarized in Figure 1. We will also tabulate each participant’s healthcare utilization and related costs using EHR at the conclusion of participation in the study protocol. We will also determine if participants are recovered (i.e., medically cleared for full return to activity) at 4 weeks and 3 months post-intervention.

### 2.6. Statistical analysis plan

All data quality monitoring and analysis will be overseen by the lead biostatistician. Distributions of baseline characteristics for participants will be compared between T-MD and usual care intervention groups to assess effectiveness of the randomization. Statistical or clinical differences will be adjusted for in secondary analyses. All analyses for treatment group comparisons will use an intention-to-treat approach and results will be reported using the CONSORT extension to non-pharmacological randomized trials. The primary outcomes for this study are NSI (Aims 1, 3), PGIC scores (Aim 1) and brain activation as measured by fNIRS (Aims 2, 3). The NSI and PGIC will be measured at baseline, 2 weeks, 4 weeks, and 3 months post randomization. Brain activation will be measured at baseline and 4 weeks. NSI and fNIRS will be treated as continuous and PGIC scores will be dichotomized into minimal or no change vs. moderate or greater change. For Aim 1, we will analyze the NSI score using linear mixed models with fixed effects for site, intervention, time, and the intervention by time interaction along with a random effect for subject to control for the correlation within person over time. We will use a generalized linear mixed model with a logit link and the same fixed and random effects for the dichotomized PGIC outcome. We will test each intervention’s effect over time using the intervention*time interaction. For Aim 2, we will calculate change scores by subtracting the fNIRS value at 4 weeks from the baseline value. We will use a two-sample t-test to compare the change scores between the intervention and control group. For Aim 3, we will calculate change scores by subtracting the NSI value at 4 weeks from the baseline value. The efficacy of the intervention will be determined based on the results for each outcome and interpreted within the domain of that specific outcome with no adjustment for multiplicity. Pearson correlation will be used to examine the relationship of change in NSI to the fMRI value at 4 weeks in the intervention group.

We also plan to explore factors (e.g., demographics, medical history, injury information, other symptoms, and impairments) that may influence the efficacy of T-MD on the outcomes of NSI, PGIC, and RTA. This will be done by focusing only on the 4-week time point and estimating within levels of each factor the intervention effect and 95% confidence intervals. We will also explore the independent effects of each treatment domain (e.g., anxiety/mood, cognitive, migraine, ocular, vestibular, sleep, autonomic) on primary and secondary clinical outcomes within the T-MD group using a mixed model with fixed effects for domain, time, and site along with a random effect for subject to control for the correlation within person over time. We will test each treatment domain’s effect over time using a domain*time interaction.

Calculations of sample size were based on the primary NSI primary outcome. We propose to randomize a total of 250 participants (125 in each intervention group) to allow approximately 200 participants for a complete case analysis at 4 weeks (assuming 20% attrition). With α = 0.05, two-tailed test, a sample size of 200 (*n* = 100 in each main effect group) will provide 80% power to detect a difference as small as 5 points on the NSI scale (standard deviation of 14) between the T-MD and usual care intervention groups assuming an autocorrelation of 0.7 for a 2 group repeated measures design with 4 repeated measures and AR (1) covariance structure. This sample size provides 99% power for the dichotomized PGIC (Aim 1) assuming 75% of the T-MD group and 50% of the control group reach moderate improvement and the same repeated measures design. Group sizes of 100 achieves 94% power to detect an effect size of 0.5 in fNIRS change using an independent samples, two-sided t-test (Aim 2, α = 0.05). For Aim 3, *n* = 100 in the intervention group provides 99% power to detect a Pearson correlation as small as 0.4. All power analyses were conducted in NCSS/PASS.

## 3. Discussion

The current study is a multisite RCT of a targeted multidomain intervention for complex mTBI in military-aged civilians. Specifically, this project will determine indicators of successful recovery using clinical profile-based outcomes and correlate these outcomes with brain function using a cost-effective, portable optical imaging tool- fNIRS. The findings from this proposal may impact the treatment of complex mTBI by utilizing empirical evidence to inform targeted interventions that build on our previous research. Deliverables from the current study will include: (1) outcome data regarding the effectiveness of T-MD treatments for complex mTBI, (2) concurrent optical brain imaging to corroborate clinical findings, and (3) evidence-based clinical practice guidelines for evaluating and treating complex mTBI.

### 3.1. Data collection, integrity, and analysis data collection, integrity, and analysis

Quality assurance will be closely monitored throughout the study and will be overseen by the study lead biostatisticians. All study team members (both researchers and clinical providers) will participate in study-related trainings, in addition to yearly refreshers. A study Manual of Operations (MOP) that identifies and outlines all study related procedures. Has been created and shared across the sites. The research team has created participant checklists for the clinical providers, specific to the study arms that assists with intervention adherence. The study will utilize a central electronic data capture system (EDC) that meets security requirements for all the study institutions with individual password access and tracking. All data entry mechanisms contain data type and value range limitations to control for extraneous data entry and missing data. Monthly and quarterly monitoring will be completed by the project manager and project coordinators from both sites to ensure timely and accurate collection and entry. When issues are identified *via* the monitoring, the site and study member will be contacted to discuss and correct any errors. Sites and their coordinators may be asked to review and verify data with the oversight of the project manager to correct data through this mechanism. Additionally, the study teams will meet regularly (at least once per month) and the coordinators will meet weekly throughout the study, to frequently discuss progress, data safety, monitoring and the integrity of the study and adherence to the study intervention.

### 3.2. Intervention compliance

Compliance with study interventions is critical to the success of this trial. We will utilize daily text messaging to ensure each participant’s compliance with their assigned intervention; and to assess symptoms and percent back-to-normal in between their initial, 2-, and 4-week in-clinic study intervals. Participants will receive an initial text message the day following enrollment into the study and a separate QR code to reinforce specifics about their assigned intervention. Inquiries about any questions they may have will be answered by email or phone call. Thereafter, daily automated text reminders will be sent each evening to remind and briefly assess compliance with the subject’s assigned intervention. The text response will take subjects to a link with four items regarding: (1) Did you perform your prescribed concussion intervention today? - yes/no for each assigned intervention (For T-MD intervention group there will be a drop down with question #1 for each T-MD intervention; CBT for anxiety/mood, cognitive accommodation/exercises, behavioral management for migraine/headache, oculomotor exercises, sleep intervention, vestibular rehabilitation, graded exertion) as determined during the adjudication process. For the usual care intervention group there will be a drop down for each of the following interventions: rest/activity, hydration, nutrition, sleep, and stress), (2) How would you describe your overall concussion symptoms today? – better, no change, worse, (3) How would you rate your percent back to normal from your injury today? 0–100% analog scale for percent back to normal, and (4) Do you have any problems, questions, or concerns that you would like to tell us at this time? - yes/no (if yes, then open dialog box to send message to the research team). Each text message will provide the participants with a globally unique identifier (GUID) link to a web page hosted through Qualtrics, a web-based data management portal to acquire and manage survey data without the need for identifying information. The Qualtrics system is HIPAA compliant, secure, and allows for easy transfer and aggregation of data that are unique to each participant. The total time to complete all three responses each day will be 2–3 min. Overall each participant will receive 28 text messages during the intervention starting at enrollment and lasting until the 4-week visit.

### 3.3. Limitations and future considerations

The current sample will likely include more women than men, which does not accurately reflect the US active-duty military population, thereby limiting the generalizability of the findings to this population. Some participants may be more or less compliant with the assigned interventions, which could limit their effect. However, we will be conducting intention to treat analyses and assessing compliance *via* text-based survey to allow for a post-hoc evaluation of the effect of treatment compliance. Some participants might engage in additional interventions outside of those assigned in the treatment arms. Although we will be assessing these adjunct treatments *via* patient self-report, we will not be able to limit or assess the effects of these interventions. Although clinical care that occurs outside of the participating health systems is rare, CPT codes for this potential occurrence will not be available to the research team and therefore not be included in the healthcare utilization analysis. Many participants will be adjudicated with multiple clinical profiles and will receive a concomitant number of concurrent targeted treatments. As such, we will not be able to connect a specific intervention with a specific treatment effect *per se*. We are also assessing compliance *via* text messaging with at-home programs that are matched to the assigned profiles and this compliance does not include measures of intensity or duration for these at home exercises (i.e., RPE), and we also acknowledge limitation of reporting bias for these self-report compliance data. Moreover, efforts will be made to keep in-clinic contact time (i.e., time and attention from clinicians) equal for the two groups. However, the time spent on completing at home interventions, as part of the assigned profile, will vary between groups due to the inherent differences between these specific interventions for the treatment group and the general recommendations for behavioral regulation assigned to the control group. In addition, the results of this trial will inform the clinical care for symptoms and impairments commonly underlie the concussion clinical profiles (e.g., headache, mood, dizziness) and may not be generalizable to other less frequent symptoms and impairments that may also occur following concussion (e.g., tinnitus, stuttering). Researchers should also consider comparing the effect of concurrent treatments for multiple profiles to prioritized treatments (i.e., for a primary profile) to better delineate and capture intervention-specific effects. Finally, although we are powered to detect group changes across time in the primary outcomes, we are not powered to detect intervention and profile (i.e., domain) specific effects across participants.

### 3.4. Anticipated outcomes

Study findings may inform a more effective approach for treating complex mTBI that minimizes morbidity and accelerates RTA/D in US military personnel, thereby decreasing the risk for long term effects on U.S. military personnel and their families. Deliverables from the current study include: (1) outcome data regarding the effectiveness and healthcare utilization costs of a targeted multi-domain intervention for complex mTBI, (2) concurrent optical brain imaging to corroborate clinical findings, and (3) evidence-based clinical practice guidelines for evaluating and treating complex mTBI. The findings may “have a significant impact on the treatment and management of complex mTBI” by informing better “clinical guidance” based on “emerging approaches” and empirical evidence for the effectiveness of targeted interventions that build on our previous and current DoD-funded research.

## Ethics statement

The studies involving human participants were reviewed and approved by the Institutional Review Board at the University of Pittsburgh. The patients/participants provided their written informed consent to participate in this study. Written informed consent was obtained from the individuals for the publication of any potentially identifiable images or data included in this article.

## Author contributions

RE, AT, and AK conceptualized the study and paper, wrote several sections of the paper, edited the entire paper, and approved the paper. MW, AM, SF, KS, CH, CD, PS, TH, DO, and MC conceptualized the study and paper, edited the entire paper, and approved the paper. KD contributed to the data analysis management, edited the entire paper, and approved the paper. CS and CP contributed to the analysis plan and sample size analysis, edited the entire paper, and approved the paper. All authors contributed to the article and approved the submitted version.

## Funding

The current study (W81XWH-20-10745) was funded by the US Army Medical Research and Materiel Command through the FY19 Psychological Health/Traumatic Brain Injury Research Award Program Complex Traumatic Brain Injury Rehabilitation Research Clinical Trial Award (W81XWH-19-CTRR-CTA).

## Conflict of interest

AK and MC receive funding for research through the University of Pittsburgh from the National Football League and royalties from APA Books. MC was a co-developer and shareholder for ImPACT applications (ended December 2019).

The remaining authors declare that the research was conducted in the absence of any commercial or financial relationships that could be construed as a potential conflict of interest.

## Publisher’s note

All claims expressed in this article are solely those of the authors and do not necessarily represent those of their affiliated organizations, or those of the publisher, the editors and the reviewers. Any product that may be evaluated in this article, or claim that may be made by its manufacturer, is not guaranteed or endorsed by the publisher.
