# Supplementary material for: Targeted multidomain intervention for complex mTBI: protocol for a multisite randomized controlled trial in military-age civilians
Source: Front Neurol. 2023 Jun 30;14:1085662. doi: 10.3389/fneur.2023.1085662 (PMC10349652; doi:10.3389/fneur.2023.1085662)
Supplement: Supplementary file 1 [file Data_Sheet_1.docx]

**Behavioral Management Strategies**

**You should engage in each of the following strategies to enhance your brain’s recovery from your concussion.**

**Sleep
Goal: Sleep your normal amount of sleep, with a goal of 8-10 hours each night.**Go to bed at the same time each night, and wake up at the same time every morning – even on weekends. Select a time that will allow you to get between 8-10 hours of sleep each night, and allow for 15-30 min of time to fall asleep each night. Avoid naps, unless napping was part of your typical schedule prior to injury. Naps can make it difficult for you to fall asleep, and lead to poor quality sleep that will prolong your recovery.

*Good sleep behavior has been shown to reduced fatigue, headache/migraine, and emotional distress.*

**Nutrition
Goal: Eat three meals throughout each day, having meals at the same time.**Eat three meals spread throughout each day including breakfast, lunch and dinner, and eat your meals at the same time each day (i.e., eat lunch at 12p every day). Aim at consuming a well-balanced diet. Eat healthy snacks in between meals. Do not skip meals, especially breakfast. Eat a well-balanced diet that is low in sugar and fats.

*Balanced and consistent nutrition will help fuel your body and support your brain’s recovery.*

**Hydration
Goal: Drink 8-10 8 oz. glasses of liquids throughout each day.**Drink water! Dehydration can lead to fatigue, headaches, dizziness, weakness, and impaired performance. Minimum number of ounces to have each day is 70-90 fluid ounces or 9-11 eight ounce cups. All fluids counts, except alcohol. Recommendation is 16-20 ounces of fluid within 1 hour of waking, 20 ounces of fluid with each meal, and 16-20 ounces with every snack. Drinks with electrolytes, such as coconut water, are an excellent source of hydration. Avoid alcohol, excessive caffeine (e.g., coffee, tea, energy drinks), and drinks that are high in sugar (e.g., soda pop, juice with added sugar) while you recover.

*Good hydration can reduce fatigue, headaches, dizziness, weakness, and enhance performance.*

**Physical Activity
Goal: Engage in 15-20 minutes of light physical activity each day.**Get some type of physical activity every day – such as walking or stationary cycling – each day. You can engage in 15-20 minutes each day. If your symptoms reach a 3/10, take a break. It’s important to remain involved in social, non-risk activities during recovering from concussion, with using breaks as needed to manage your symptoms.

*Physical activity can enhance recovery from concussion, BUT monitor your symptoms and take breaks when needed.*

**Stress Regulation
Goal: Engage in stress regulation strategies each day.**

Both physical and emotional stress can cause and/or worsen your symptoms. Your doctor may provide you with recommended school/work accommodations to reduce stress while you are healing, and it is important to follow these recommendations. In addition, you should engage in the following stress regulation strategies each day: 1) be aware of your situational and environmental stressors (e.g., thinking about your injury, isolating yourself); 2) maintain a consistent routine or schedule (e.g., meals, sleep, school, practice) and electronics (e.g., cell phone, video games, TV) can be used for 20-30 minute periods, followed by a break; 3) be socially active (e.g., attend – but do not participate in- team practices, spend time with friends/family, use technology to stay connected), and 4) engage in coping strategies when stressed (listening to music, relaxing, etc.).

*Engaging in regular stress regulation is an important way to help improve your symptoms.*
