# Supplementary material for: Targeted multidomain intervention for complex mTBI: protocol for a multisite randomized controlled trial in military-age civilians
Source: Front Neurol. 2023 Jun 30;14:1085662. doi: 10.3389/fneur.2023.1085662 (PMC10349652; doi:10.3389/fneur.2023.1085662)
Supplement: Supplementary file 2 [file Data_Sheet_2.docx]

**Primary Outcomes.**

*Demographics and Medical History.* Participants’ age, sex, height, weight, BMI, occupation, current work/study status, and years in current position will be recorded. Recent medical history including history of mTBI, ADHD/LD, migraine, motion sickness, and medication use will be obtained from electronic health records (EHRs). Injury information including the current symptoms and complaints, mechanism of injury (sport-related, motor vehicle collision, assault, falls), description of symptoms (intensity, location, nature), numeric headache and bodily pain rating scales, sleep hygiene, and aggravating and mitigating factors will be recorded. Social history is examined with documentation of living situation and recreational activities.

*Neurobehavioral Symptom Inventory (NSI)*. The NSI is a 22-item (i.e. symptom) scale in which participants rate the severity of symptoms on a 5-point scale (0 = None, 1 = Mild, 2 = Moderate, 3 = Severe, 4 = Very Severe) ranging from 0-60 (King et al., 2012).

*Patient Global Impression of Change (PGIC).* The PGIC was developed for self-reported assessment of change resulting from post-mTBI care at military treatment facilities. The prompt is: “Since beginning treatment at this facility, how would you describe the change (if any) in activity limitations, symptoms, emotions and overall quality of life related to your post-concussive condition?” The patient responds on a scale ranging from 0 (No change or condition has gotten worse), to 7 (A great deal better and a considerable improvement that has made all the difference) (Hurst et al., 2004).

*Functional Near-infrared Spectroscopy (fNIRS).* Brain Activation/Deactivation fNIRS is a non-invasive optical imaging tool that measures changes in cerebral blood flow (CBF) and oxygenation and allows for imaging during movement with limited noise. The fNIRS signal corresponds to the BOLD signal from fMRI (Huppert et al., 2006). fNIRS involves low levels of visible red and near-infrared light (<0.4 W/cm^2^) that penetrate approximately 5-8mm into the cerebral cortex via source emitters on the scalp (Fig 8) (Boas et al., 2005). Source detectors then record the changes in the amount of red and near-infrared light that is absorbed and refracted to determine blood volume and concentrations of oxygenated and deoxygenated hemoglobin. During the performance of a task or following brain injury, regional changes in oxygenated and deoxygenated hemoglobin concentration alter absorption of light in the brain, which is then measured and quantified. fNIRS will be employed during two different paradigms: 1) at rest, and 2) during cognitive activity (i.e., congruent/incongruent flanker task).

**Secondary Outcomes**

*Behavioral Symptom Inventory (BSI-18).* The BSI-18 is an 18-item symptom inventory that assesses the level of psychological distress during the past 7 days on 18 items. The BSI-18 yields total global severity index ranging from 0-72, as well as somatic, depression, and anxiety sub-scale scores. Any sub-scale T-score >63 is reflective of clinical impairment on that sub-scale. The BSI-18 requires 5 minutes to complete and score (Derogatis, 2001).

*Dizziness Handicap Inventory (DHI).* The DHI is a 25-item self-report measure that examines dizziness-related handicap (Jacboson 1990). Each item is categorized into one of three domains: functional, emotional, or physical. It was developed with the help of patients who complained of dizziness and uses a three-item response scale, “yes/sometimes/no” scored as “4/2/0” respectively. The DHI has good internal consistency for the total score (a = 0.89) (Jacobson, 1990). The test-retest reliability is high (r = .97) and it is responsive to change in a vestibular population. The DHI have been demonstrated to be valid in individuals with mTBI. The DHI takes approximately 5 minutes to complete (Jacobson et al., 1990).

*Vestibular/Ocular Motor Screening (VOMS).* The VOMS will be used to screen for vestibular and oculomotor symptoms and impairment. The VOMS assesses impairment via patient-reported symptom provocation following each of the following components: 1) smooth pursuits, 2) horizontal and vertical saccades, 3) convergence, 4) horizontal and vertical vestibular ocular reflex (VOR) and 5) visual motion sensitivity (VMS). Patients verbally rate changes in headache, dizziness, nausea and fogginess symptoms compared to their immediate pre-assessment state on a scale of 0 (none) to 10 (severe) following each VOMS assessment, to determine if any domain provokes symptoms. A change score (relative to pretest symptom score) > 2 on any VOMS item reflects a positive screening cut-off for vestibular or oculomotor impairment (Mucha et al., 2014). Convergence is also assessed in the VOMS using both symptom report and objective measurement of the near point of convergence (NPC averaged across 3 trials). NPC values >5 cm reflect a positive clinical screening cut- off (Pearce et al., 2015). The VOMS requires limited equipment: a 14 pt font NPC hand-held fixation stick, metronome, and a 1-page paper scoring form. The VOMS requires approximately 5 minutes to administer and score (Mucha et al., 2014).

*Modified Balance Error Scoring System (mBESS).* The mBESS measures postural stability and consists of three stances including feet side by side, a tandem stance, and a single-leg stance on the non-dominant leg (Guskiewicz et al., 2001) The three stances are performed for 20 sec each, three on a firm surface and three stances on a dynamic (medium density foam) surface. All stances are completed with eyes closed and with hands on the iliac crests. Errors include lifting hands off the iliac crests, opening the eyes, stepping, stumbling, or falling, moving the hip into more than a 30 degree of flexion or abduction, lifting the forefoot or heel, or remaining out of the testing position for more than 5 seconds. Each error equals 1 point, with higher scores indicating worse performance. For the current study, we will use the modified BESS (mBESS) that consists of the three stances performed on the firm surface only. Clinical cut-offs for BESS suggest that a total error score 9 or greater indicates clinical impairment in balance (Covassin et al., 2016). The mBESS takes approximately 5-6 minutes to administer (Iverson et al., 2013).

*Functional Gait Assessment (FGA).* The 10-item FGA assesses the ability of participants to walk with head turns, changes of speed, and around obstacles. Each item is scored on a 4-point ordinal scale ranging from 0- severe impairment to 3- normal Higher scores indicate normal gait. The FGA takes 5-10 minutes to complete (Wrisley et al., 2004).

*Clinical Profile Screening Inventory (CP-Screen).* The CP-Screen is a 29-item self-report, clinical profiles-based symptom inventory that measures five concussion clinical profiles: 1) anxiety/mood, 2) cognitive/fatigue, 3) migraine, 4) ocular, 5) vestibular; and two modifying factors: 1) sleep, 2) neck. Participants indicate on a scale of 0-none to 3-severe the level of symptom severity for each item. The CP-Screen yields average factor and modifier scores, with higher scores indicative of worse symptom severity. The CP-Screen takes 3-5 minutes to complete (Kontos et al., 2019).

*Immediate Post-concussion Assessment and Cognitive Testing (ImPACT).* The ImPACT will be used to assess neurocognitive performance in civilians. ImPACT is a computerized neurocognitive test that includes six modules: 1) verbal memory, 2) design memory, 3) X’s and O’s, 4) symbol matching, 5) color matching, and 6) three letter memory (ImPACT Applications Inc.) (Lovell et al., 2006). These modules are used to form four composite scores: verbal and visual memory (%), visual motor processing speed (#), and reaction time (RT) (sec). The ImPACT test takes 20-30 minutes to administer.

*Pittsburgh Sleep Quality Index (PSQI).* The PSQI will be used to assess sleep quality. The PSQI is a self-report measures including 18 items that comprise seven component scores: 1) subjective sleep quality, 2) sleep latency, 3) sleep duration, 4) sleep efficiency, 5) sleep disturbances, 6) sleep medication usage, and 7) daytime dysfunction (Buysse et al., 1989). Subscale and global PSQI scores are calculated, with higher scores indicating poorer sleep quality. The PSQI takes 2-3 minutes to complete (Carpenter et al., 1998).

*Headache Impact Test (HIT-6).* The HIT-6 comprises six self-report items that assess frequency of severe headache, limitations of daily activities, desire to lie down, fatigue, irritability, and difficulty concentrating (Nachit-Ouinekh et al., 2005). Individual items scores range from 6-never to 13- always. Overall severity scores range from 36-78, with higher scores indicative of worse severity. Grades of total HIT-6 scores include: no impact (score range= 36-49), moderate (score range= 50-55), substantial (score range= 56-59), or severe impact (score range= 60-78). The HIT-6 takes 2-3 minutes to complete.

*ID Migraine.* The ID Migraine will be used to screen for migraine headache symptoms. The ID Migraine is a 3-item screening tool designed to assess presence (yes/no) of symptoms related to headache/migraine pain including nausea, sensitivity to light, and functional impact of headaches. Scores range from 0-3 with clinical cut off of 2+ indicating presence of migraine (Lipton et al., 2003). The ID Migraine is both a valid and reliable screening tool for migraine with good, combined sensitivity and specificity (Lipton et al., 2003). The ID Migraine requires 2 minutes to complete and score.

*Short-form McGill Pain Questionnaire (SF-MPQ).* The SF-MPQ is a 15-item pain scale in which participants rate the intensity of their pain on a 4-point scale (0= None, 1=Mild, 2=Moderate, 3=Severe) (Wright et al., 2001). Outcomes are total score, as well as sensory and affective pain subscale scores. SF-MPQ takes 2-3 minutes to complete.

*International Physical Activity Questionnaire (IPAQ).* The International Physical Activity Questionnaire (IPAQ) will be used to assess potential treatment group differences in activity level (Craig et al., 2003). The IPAQ is a validated tool to assess recall of average activity level and intensity over the preceding 7 days. The IPAQ will be completed via text/online survey at 7, 14, 28, days and 3 months. It was chosen because it is brief and can be either self-administered or administered via structured phone interview. Activity levels can be expressed as categorical variables (low, medium, high activity levels) or a continuous variable (MET-minutes/week). The IPAQ takes approximately 5 minutes to complete.

*Buffalo Concussion Bike Test (BCBT).* The BCBT will be utilized to measure autonomic dysfunction following mTBI (Leddy et al., 2011). Prior to test initiation, resting heart rate (HR) is measured after a 2-minute seated resting period. A visual analog scale is used to rate symptoms at baseline. The participant starts the test by pedaling at 60 + 5rpm, which is the speed that must be maintained during the test. After acclimating to this pace for 2 min, power output is adjusted based on the protocols Weight to Power/Watt Conversion Table every 2 min for the next 30 min. Outcomes are HR threshold, visual analog scale, and Borg Rating of Perceived Exertion measured every 2 min until symptoms are exacerbated or voluntary exhaustion is reported. Symptom exacerbation is defined as an increase of 3 or more points on the pre-exercise Visual Analog Scale value. Voluntary exhaustion is defined as a 17 or more on the Rating of Perceived Exertion scale. HR threshold is the participant’s HR at time of symptom exacerbation or time voluntary exhaustion is reported. The BCBT takes approximately 5-30 minutes to complete depending on symptom provocation during the test.

*Text Message-based Compliance Assessment.* We will utilize daily text messaging to ensure each participant’s compliance with their assigned intervention; and to assess frequency and intensity of at-home exercises in between the initial, 2-, and 4-week in-person study intervals. We have used similar systems previously with between 85-90% response rate in patients with concussion (Snook et al., 2017). Participants will receive an initial text message immediately following enrollment into the study. A separate QR code is given to the participant with specific information about their assigned intervention and information to contact the study team about any questions they may have that will be answered by email or phone call. Thereafter, daily automated text reminders will be sent each evening to remind and briefly assess compliance with the subject’s assigned interventions. The text response will take participants to a link with items for each assigned intervention regarding 1) compliance yes/no; 2) overall symptoms- better, no change, worse; 3) percent back to normal participant’s feel each day (0-100%), and 4) problems, questions or concerns participants may have about their intervention or assigned exercises. In total, each of the 250 subjects enrolled in the study will be sent text messages 28 times across the first 4 weeks of the study. Therefore, we expect to send and receive up to 14,000 text messages for the study period across all participants. Each text message will provide the participants with a globally unique identifier (GUID) link to a web page hosted through Quesgen, a web-based data management portal to acquire and manage survey data without the need for identifying information. The Quesgen system, is HIPAA compliant, secure, and allows for easy transfer and aggregation of data that are unique to each participant. The total time to complete the daily surveys will be 2-3 minutes each day.
