# Supplementary material for: Targeted multidomain intervention for complex mTBI: protocol for a multisite randomized controlled trial in military-age civilians
Source: Front Neurol. 2023 Jun 30;14:1085662. doi: 10.3389/fneur.2023.1085662 (PMC10349652; doi:10.3389/fneur.2023.1085662)
Supplement: Supplementary file 3 [file Table_1.docx]

Supplemental Material Table 1. Description of interventions for T-MD

| **Domain** | **Recommended Treatments/Interventions** | |
| --- | --- | --- |
| Anxiety/  Mood | If patients are adjudicated to have anxiety/mood, the intervention will involve one or more of the following strategies:   1. Engage in Physical Activity 2. Engage in Social Activity 3. Follow a Consistent Daily Schedule 4. Stress Management - Deep Breathing 5. Stress Management - Muscle Relaxation 6. Stress Management – Meditation 7. Compliance   Subjects will receive written instructions to direct this intervention, which include specifications for how much time should be spent on each activity per day.  **Engage in Physical Activity**  Subjects will be instructed to engage in non-contact cardiovascular activity 5-7 days per week, slowly increasing duration and intensity.  **Engage in Social Activity**  Subjects will be instructed to engage in regular social activity with friends and family each day; examples of social activities are provided in the handout.  **Follow a Consistent Daily Schedule**  Subjects will be instructed on how to form and maintain a consistent daily schedule to create a framework for recovery. Subjects are encouraged to spend 5-10 minutes each day updating and reviewing their schedule.  **Stress Management Strategies**  Deep breathing, muscle relaxation, and meditation are all mindfulness-based exercises that the subjects can use to promote a relaxation response. Subjects will be provided with detailed instructions to guide them through these exercises. If assigned the Muscle Relaxation or Meditation strategies, the subject will also be provided with an audio recording to accompany the intervention.  **Compliance**  Compliance with specific anxiety/mood interventions will be collected via the daily text messaging surveys. Daily automated text reminders will be sent each evening to remind and briefly assess compliance with the subject’s assigned intervention. The text response will take subjects to a link with four items regarding:   1. Did you perform your assigned ANXIETY/MOOD concussion intervention today? **YES/NO** 2. How would you rate your overall concussion symptoms today? **BETTER/NO CHANGE/WORSE** 3. How would you rate your percent back to normal from your injury today? **0-100% analog scale** 4. Do you have any problems, questions or concerns that you would like to tell us at this time? **YES/NO** | |
|  |  |  |
|  |  |  |
| Cognitive | If patients are adjudicated to have cognitive, the intervention will involve one or more of the following strategies:   1. Academic accommodations 2. Work restrictions 3. Goal setting. 4. Compliance   Participants will be assigned academic accommodations/work restrictions based on their specific impairments and symptoms. These accommodations are described below. Each accommodation will be assigned as indicated for specific issues (i.e., ocular symptoms, vestibular symptoms, migraines/headaches, anxiety management) that could interfere with the patient’s ability to perform work or academic requirements as normally expected. Accommodations may include (dependent upon if used for work/school and specific issues as outlined above) use of rest breaks during cognitive activities, schedule modifications, job modifications, extended deadlines, note taking and extended test-taking time. Accommodations will be recommended to be used as needed, though if breaks are recommended a minimum amount of stimulus breaks (mid-morning, lunch time and mid-afternoon) for 10-15 minutes will be recommended. In addition, participants will be guided in proper goal setting to help prioritize and manage activities. See below for more details about each component:  **Academic (if patient is returning to an academic environment)**  **Testing Accommodations:** Students with concussions may experience cognitive difficulties, such as difficulty remembering and concentrating. Highly demanding activities like testing can increase symptoms (e.g. headache, fatigue) which in turn can make testing more difficult. Therefore, recommendations may include **extra time (recommended at 50%), testing in a quiet environment, and/or enlarged font and/or reduced items on a page**.  **Note Taking:** Note taking may be difficult after concussion due to difficulty multitasking and increased symptoms. **Please allow this student to obtain class notes or outlines ahead of time to aid organization and reduce multi-tasking demands.**  **Extra Time:** Students may experience increased symptoms throughout the day or after the school day while recovering from a concussion. Therefore, this student may need to turn in assignments late on occasion. **Please allow extra time (to be worked out individually with instructor) to complete and turn in assignments.**  **Work (if patient is returning to a work environment)**  **Schedule Modifications:** Schedule modifications can assist an individual in successfully returning to the workplace following concussion by allowing the patient to gradually work up to prior number of hours per week. **This may include the recommendation for completion of half days or reduced hours (compared to normal schedule) for a brief period of time, with the goal being resuming a normal schedule as soon as possible.**  **Job Modifications:** Job modifications for some professions are necessary to help an individual successfully return to the workplace following concussion by allowing the individual to gradually increase task levels and work towards previous job requirements. **This may include the recommendation for completion of light duty tasks or elimination of a specific role (e.g., a task that may exacerbate symptoms) for a brief period of time, with the goal being resuming a normal schedule as soon as possible.**  **Extended Deadlines:** Individuals with a concussion may experience increased symptoms throughout the day while recovering from a concussion. Therefore, this individual may need extended deadlines on occasion. **Please allow extra time (to be worked out individually with supervisor) to complete and turn in assignments.**  **Goal Setting**  The primary objective of goal setting is to help individuals break down larger tasks into smaller and manageable components to help facilitate completion of successful events during the recovery process. It is well known that many individuals struggle to complete tasks in the manner they did prior to the concussion while still recovering. Therefore, helping individuals learn to set reasonable and attainable goals is essential in some cases. Principles of goal setting will be reviewed individually with the patient and a goal setting worksheet (following the SMART philosophy) will be provided to the individual to complete that can then be shared with the clinical staff during follow-up appointments. Participants will be given the S.M.A.R.T. goal worksheet (see figure below) to list their goals and track progress towards their identified goals.  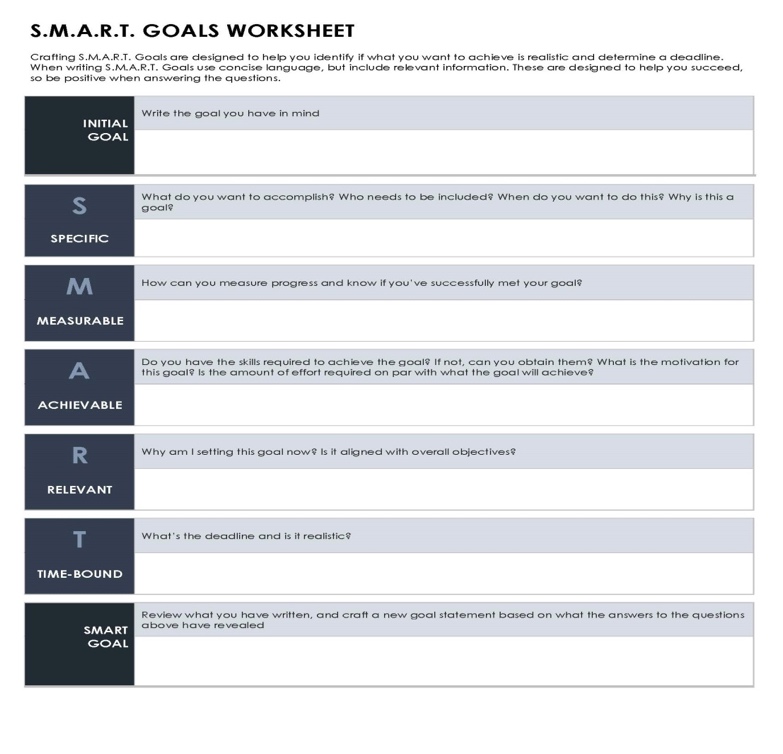  **Compliance**  Compliance with specific cognitive interventions will be collected via the daily text messaging surveys. Daily automated text reminders will be sent each evening to remind and briefly assess compliance with the subject’s assigned intervention. The text response will take subjects to a link with four items regarding:   1. Did you perform your assigned COGNITIVE concussion intervention today? **YES/NO** 2. How would you rate your overall concussion symptoms today? **BETTER/NO CHANGE/WORSE** 3. How would you rate your percent back to normal from your injury today? **0-100% analog scale** 4. Do you have any problems, questions or concerns that you would like to tell us at this time? **YES/NO** | |
|  |  |  |
| Migraine/ Headache | If participants are adjudicated to have migraine/headache, the intervention will involve one or more of the following strategies:   1. Regulating Sleep 2. Balancing Nutrition 3. Staying Hydrated 4. Regulating Stress 5. Engaging in Physical Activity 6. Sensitivity Training – for Migraine Symptoms Only 7. Statement about Over-the-Counter (OTC) Medications and Supplements 8. As with all intervention strategies, subjects will receive written handouts elaborating on these topics after the clinicians first discuss them with the subject.   .  **Regulating Sleep**  Subjects will be given guidelines for general sleep regulation, which includes directives for going to bed at the same time each night, waking at the same time each morning, avoiding technology use 15-30 minutes before bed, setting 15-30 minutes of time to fall asleep each night, etc.  **Balancing Nutrition**  Subjects will be given some tips for engaging in balanced nutrition throughout the day, which include eating 3 well-balanced meals spread throughout the day, eating meals at the same time each day, avoiding fasting for longer than 3 hours, etc.  **Staying Hydrated**  Subjects will be instructed to drink at least 60 fluid ounces each day, limit drinks with alcohol, artificial sweeteners, and excessive caffeine, and to not limit nor increase the daily caffeine intake that the subject had previously engaged with, pre-injury. Drinks with electrolytes may be encouraged.  **Regulating Stress**  Subjects may be provided with school/work accommodations to help regulate stress during recovery. Additional stress regulation strategies recommended may include cultivating awareness of situational and environmental stressors, maintaining a consistent routine, being socially active, engaging in coping strategies when stressed, and engagement with other, individualized techniques and therapies.  **Engaging in Physical Activity**  Subjects will be encouraged to increase non-contact physical activity. Subjects will be instructed to, at first, exercise to a mild to moderate level of symptoms and to take a break before symptoms reach a severe level ( i.e. >5 of 10). Subjects will be instructed to slowly increase the duration and intensity of their physical activity, until exercise is a regular habit of 30-60 minutes per day.  **Sensitivity Training for Migraine Symptoms Only**  For subjects with migraine symptoms, this intervention provides specific suggestions for light, noise/sound, and motion sensitivities. Subjects will be instructed to expose themselves to stimuli that are somewhat bothersome in order to help their brain adjust and accommodate the aggravating stimuli.  **Statement about Over-the-Counter (OTC) Medications and Supplements**   - Common OTC headache medications including ibuprofen (Advil), acetaminophen (Tylenol), naproxen (Alleve), and Excedrin migraine should be used no more than 2 days per week to avoid a rebound or “medication-overuse” headache. Do not use OTC medication more than 2 days unless directed to do so by your treating clinician. - Supplements, such as magnesium, vitamin B2 (riboflavin), and coenzyme Q-10 (Co-Q10) have been shown to help reduce migraine episodes and may also be taken as prescribed by your clinician. Be sure to check with your treating clinician to determine if these medications and supplements are recommended in your case.   **Compliance**  Compliance with specific migraine/headache interventions will be collected via the daily text messaging surveys. Daily automated text reminders will be sent each evening to remind and briefly assess compliance with the subject’s assigned intervention. The text response will take subjects to a link with four items regarding:   1. Did you perform your assigned MIGRAINE/HEADACHE concussion intervention today? **YES/NO** 2. How would you rate your overall concussion symptoms today? **BETTER/NO CHANGE/WORSE** 3. How would you rate your percent back to normal from your injury today? **0-100% analog scale** 4. Do you have any problems, questions or concerns that you would like to tell us at this time? **YES/NO** | |
|  |  |  |
|  |  |  |
| Ocular | If participants are adjudicated to have ocular, the intervention will involve one or more of the following ocular categories:   1. Vergence 2. Accommodation 3. Smooth Pursuits 4. Saccades 5. Compliance   Participants will be assigned one or more ocular exercises based upon their specific impairments and symptoms. These exercises are described below. Each oculomotor rehabilitation exercise will be assigned as indicated for specific dysfunction (i.e., ocular symptoms [headache while reading, blurry vision, double vision], near point of convergence, positive fusional vergence, accommodative amplitude). Interventions may include Brock string, pencil push-ups, saccade tracking, and pursuits). Exercises will be performed 1-2 times a day for a total of 15-20 minutes. Participants will return bi-weekly to their vestibular physical therapist for follow-up visits, where participant’s impairments and progress will be re-assessed, and exercises will be adjusted accordingly. When appropriate, participants with ocular issues may also be referred to vision therapy.  Compliance with specific ocular exercises/interventions will be collected via the daily text messaging surveys. Daily automated text reminders will be sent each evening to remind and briefly assess compliance with the subject’s assigned intervention. The text response will take subjects to a link with four items regarding:   1. Did you perform your assigned OCULAR concussion intervention today? **YES/NO** 2. How would you rate your overall concussion symptoms today? **BETTER/NO CHANGE/WORSE** 3. How would you rate your percent back to normal from your injury today? **0-100% analog scale** 4. Do you have any problems, questions or concerns that you would like to tell us at this time? **YES/NO** | |
|  | **Ocular Push Ups** (Reps: 10, 1-2 times daily)   - Focus on a target at an arms length away. - Slowly bring the target toward your nose, maintaining your focus. - Try to keep the target single (not double) as you bring it closer - Hold for 5 seconds at nearest point, then return slowly back to starting point. Try to bring the target closer every time, while keeping it single and as clear as possible   If you normally wear glasses when you read, wear your glasses when doing this exercise  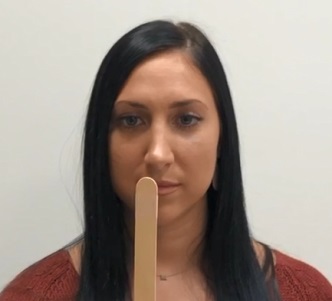 | **Hart Chart** (Reps 1-3, 1-2 times daily)   - Place the larger chart on the wall about 10 feet away and hold the smaller chart in your hand at an arms length away. - Look at the distant chart, focus and read the top line. - Look at the near chart, focus and read the top line. - Switch back to the distant chart, focus and read the second line. - Switch to the near chart, focus and read the second line. - Continue switching back and forth between the distant and near charts, reading one line down each time until you reach the bottom lines. - GRADUALLY move the near chart closer as long as you can clear and read the lines   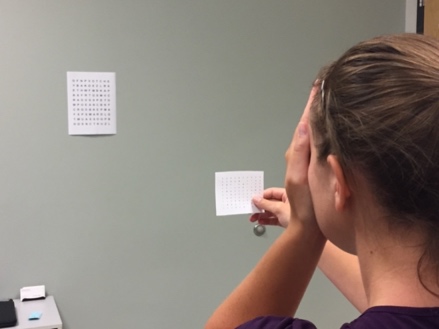 |
|  | **3 Bead Brock String** (3 Minutes, 1-2 times daily)   - Loop one end of the string to a doorknob or other solid object. Place the knot at the other side of the string on the tip of your nose and look down the length of the string. - Place the first bead at the closest point the bead remains single. - Place the second bead in the middle of the string and the third bead at the end of the string. - Focus your eyes on the farthest bead. You should see a single bead, and a “V” (opening towards you) formed by the string. Hold for 5 seconds. - Focus your eyes on the middle bead. You should see a single bead, and an “X” formed by the string. Hold for 5 seconds. - Focus your eyes on the closest bead. You should see a single bead, and a “V” (opening away from you) formed by the string. Hold for 5 seconds. - Continue to move your gaze from bead to bead for 3 minutes total, taking breaks as needed to keep your symptoms in control. - As you are able, gradually move the closest bead toward your nose 1 cm at a time.   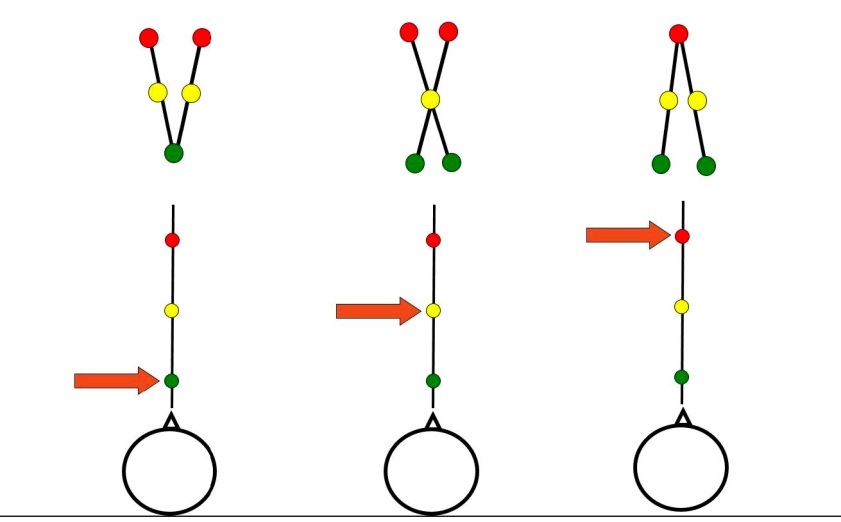 | **6 Bead Brock String** (3-5 minutes, 1-2 times daily)   - Loop one end of the string to a doorknob or other solid object. Place the knot at the other side of the string on the tip of your nose and look down the length of the string. - Adjust first bead 1 inch from your nose then space the adjacent 3 beads 1 inch behind. Space the last two beads at the middle and end of the string. - Focus on bead furthest away for 5 seconds, then middle bead, then each closer bead. Try to visualize a "V" or "X" formed by the string at each bead. - Perform for 3-5 minutes, taking breaks as needed to manage symptoms   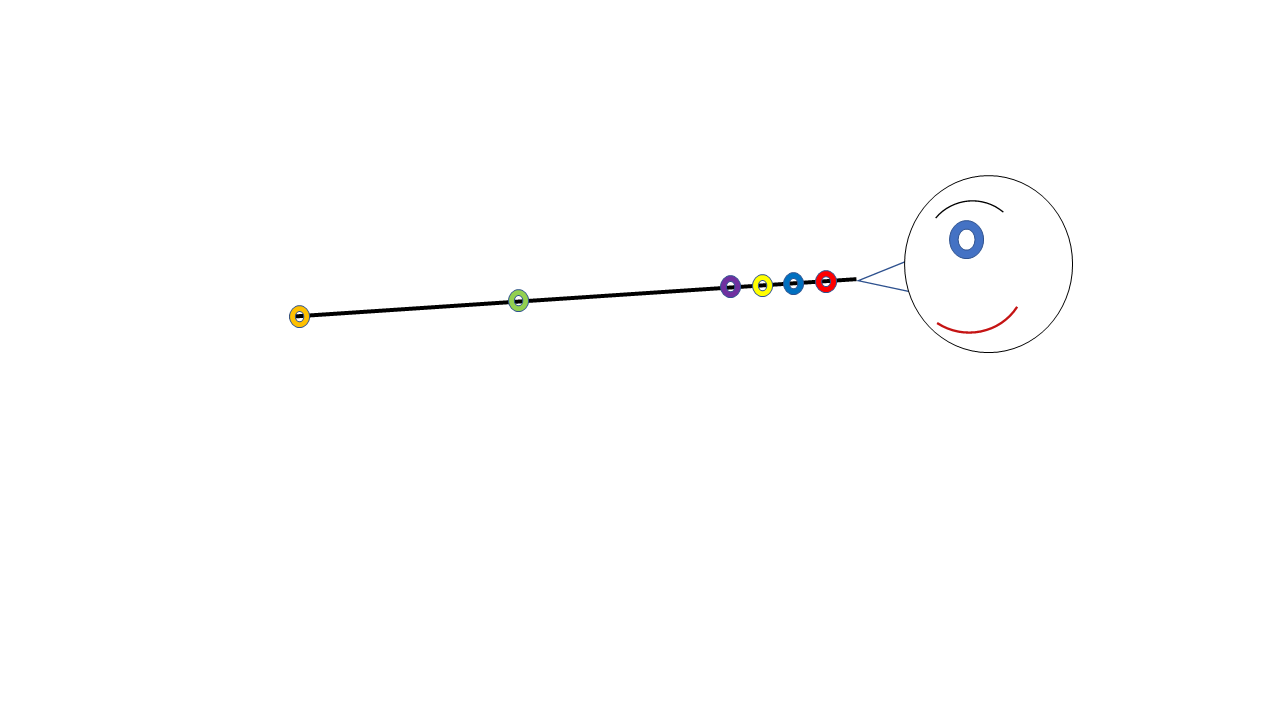 |
|  | **Dot Line:** (Reps: 3, Sets: 3, 1-2 times daily)   - Place DOT LINE on book or other flat surface. Hold in front of your nose - touching the front of the line to your nose. - Focus on dot farthest away. Try to visualize upside down "V" with lines - hold for 5 seconds. - Move to the next closest dot. Try to see the "X" with the lines. - Keep moving up the line, as long as you can keep each dot SINGLE and form an "X" with the line. - Try to work up to getting to the closest 1-2 dots (or as close to your nose as possible); then slowly reverse back to starting position.   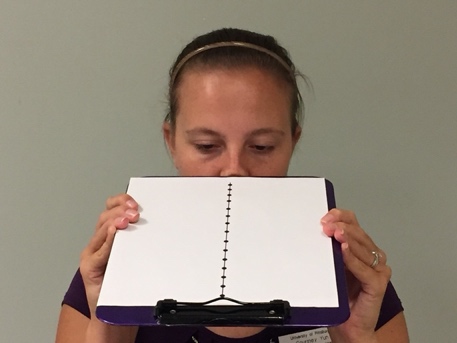 | **Smooth Pursuits** (Reps: 10, Sets: 2, 1-2 times daily)   1. Focus on a target held in your hand. Slowly move the target from SIDE TO SIDE as you maintain focus on it. Repeat and move target UP AND DOWN. 2. Repeat 10 times in each direction, 2 sets.   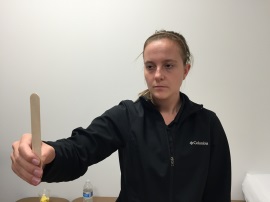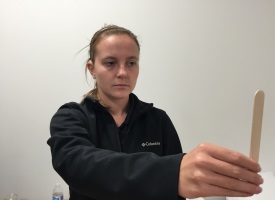 |
|  | **Saccades 4 Target on Wall** (Reps: 10, Sets: 2, 1-2 times Daily)   - Post 4 targets on a wall (2 horizontal and 2 vertical) and stand approximately arms length away. - Move your eyes as quickly as possible between the two horizontal targets. - Repeat, moving your eyes between the two vertical targets. - Repeat, moving your eyes to each target in a clockwise direction. - Repeat, moving your eyes to each target in a counterclockwise direction.   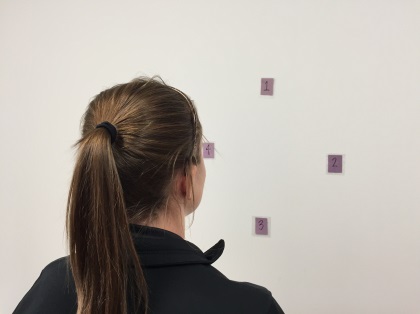 | **Saccades Letters/Numbers** (Reps: 2, 1-2 times daily)   - Post 4 pages of numbers/letters on the wall. Stand approximately arms length away. - Move your eyes as quickly as possible from 1-20. - Repeat, moving your eyes as quickly as possible from A to Z. - Rotate the order of the pages every other day.   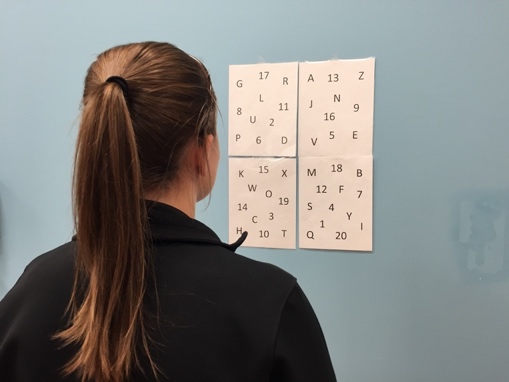 |
| Vestibular | If participants are adjudicated to have vestibular, the intervention will involve one or more of the following vestibular exercises:   1. Gaze stability for vestibulo-ocular reflex (VOR) dysfunction 2. Visual-vestibular habituation exercises for visual motion sensitivity (VMS) 3. Balance exercises for standing balance or gait dysfunction.   During the course of the intervention, we expect that the participants will progress to a stage of higher level exertional and functional performance tasks. Exercises will be performed 1-2 times per day for a total of 15-20 minutes. Participants will return bi-weekly to their vestibular physical therapist for follow-up visits, where participant’s impairments and progress will be re-assessed, and exercises will be adjusted accordingly.  1. Initial exercise level will be established (Level 1, 2, 3 or 4) at Visit 1 by the treating vestibular physical therapist.  2. Patients will be instructed to wait for symptoms to recover to baseline between each exercise set  3. Exercise Level will be increased or decreased to appropriate Level at Visit 2, based on ability to complete assigned Level  **Gaze Stability**  The primary goals of gaze stability exercises are to adapt the VOR and habituate dizziness symptoms to head movements. Therefore, the main exercise of this group is the VORx1 (i.e. VOR times 1), which is designed to produce eye movement velocities that are equal (x1) and opposite to the head movements. This exercise is performed by having the patient fixate a stationary target while they move their head back and forth horizontally or vertically. A metronome may be used to pace the movements. The amplitude of head movements are no more than 15-20 degrees from midline so that the eye movements do not reach end range. If the patient has very strong symptoms or profound impairments initially, the physical therapist may begin with head stationary oculomotor exercises such as smooth pursuits or saccades.  The exercises can be made more difficult by the following strategies. The exercises are listed in order of increasing difficulty, as recommended in the exercise progression framework of Klatt et al., (2015) [2].   - Increasing duration of movements (20 sec to 60 sec) - Increasing the frequency and thus speed of head movement (0.5 Hz to 2.0 Hz) - Making the environment more distracting (changing from a quiet room with plain visual background to a noisy gym with complex visual patterns) - Reducing base of support (wide base to narrow base) or performing on a foam surface - Adding whole body movements (standing to walking to jogging) - Moving the target (VORx2: target moves opposite to head movement, and random movements)   A typical sample progression of gaze stability exercises is provided in the following example. As can be seen, the patient starts with the VORx1 exercise with a plain background at comfortable speed for 20 sec. In week 2, the speed and duration is increased. In week 3, speed and duration are increased again and a busy background is introduced. In week 4, whole body stepping movements are added. Note that this example is just provided for the purpose of illustration, and that the PT may use any of the strategies listed above to increase the difficulty so that the intensity of the exercises is equal to that assigned for the intervention.   \| Exercise Category \| Level 1 \| Level 2 \| Level 3 \| Level 4 \| \| --- \| --- \| --- \| --- \| --- \| \| Gaze stability \| VORx1  comfortable speed  20 sec  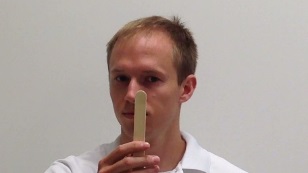 \| VORx1  120-155 bpm  30 sec  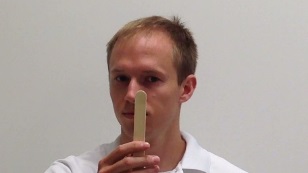 \| VORx1  160-180 bpm  30 sec  Busy background  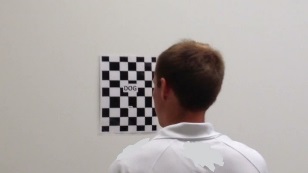 \| VORx1  180-200 bpm  30-60 sec  Busy environment  Stepping forward and backward   \|   **Visual-Vestibular Habituation**  The primary goals of visual habituation exercises are to habituate dizziness symptoms to head movements in provocative visual environments. The most simple example of this is performing the head movements while fixating a head-fixed target (i.e. VOR cancellation). This exercise is performed by having the patient hold a target at outstretched arms-length and rotating the trunk and head back and forth horizontally for a period of 20 to 60 s. A metronome is used to pace the movements ranging from frequencies of 0.1 Hz (12 bpm on a metronome if a beep occurs at each end of the head movement) up to 0.5 Hz (60 bpm). The amplitude of trunk and head movements is about 45 deg from midline.  The exercises can be made more difficult by the following strategies.   - Increasing duration of movements (20 s to 60 sec) - Increasing the frequency and thus speed of head movement (0.1 Hz to 0.5 Hz) - Making the environment more distracting (changing from a quiet room with plain visual background to a noisy gym with complex visual patterns) - Using optokinetic stimuli such as videos showing on large field of view monitors or full field optokinetic stimuli (e.g. disco ball), or viewing videos on a monitor. A list of URLs will be provided to the patients. - Locating and tracking targets while moving, such as when catching a ball from behind one’s back - Adding whole body movements (standing to walking to jogging)   A typical sample progression of visual habituation exercises is provided in the following example. As can be seen, the most basic exercise involves the VOR cancellation exercise with a plain background at slow speed for 2 sets if 10 repetitions in the horizontal and vertical directions. In week 2, the patient must track moving targets while moving head and trunk. In week 3, the subject is asked to perform an activity a busy or moving visual background. In week 4, the tracking movements are performed while walking forward and backward. Note that this example is just provided for the purpose of illustration, and that the PT may use any of the strategies listed above to increase the difficulty so that the intensity of the exercises is equal to that assigned for the intervention.   \| Exercise Category \| Level 1 \| Level 2 \| Level 3 \| Level 4 \| \| --- \| --- \| --- \| --- \| --- \| \| Visual Habituation \| VOR cancellation  Slow pace  2 sets of 10  Plain background   \| VOR cancellation  Faster Speed  3 sets of 10-15  Busy background  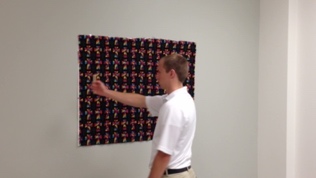 \| Backward Ball Toss with a partner  Busy environment  4 sets of 10  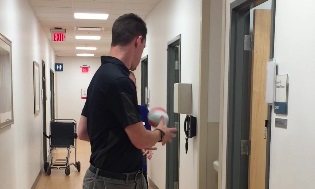 \| Backward ball toss while walking forward and backward  Fast speed  4 sets of 60 sec  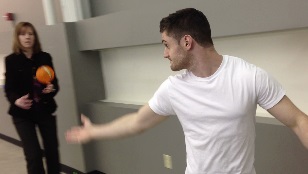 \|   **Balance**  Balance exercises are divided into three main categories: 1) standing balance 2) dynamic balance, and 3) gait.  **Standing Balance**  The primary goals of standing balance exercises are to promote sensory integration by altering the sensory input and to challenge the postural control system by changing the biomechanical demands. Sensory input is altered by closing eyes, standing on foam or uneven surfaces, or by stimulating the vestibular system with head movement. Biomechanical demands are increased by reducing the base of support.  In general, static standing balance exercises are progressed by first closing eyes, and then moving the head in the yaw and pitch planes. These same exercises are next progressed to a foam surface with feet apart. After successfully performing the exercises on foam, the base of support should be decreased to feet together, semi-tandem and tandem, starting on a firm surface and then progressing to foam. Within each surface the visual input and head movement can be modified to provide additional challenges.  The exercises can be made more difficult by the following strategies. The exercises are listed in order of increasing difficulty, as recommended in the exercise progression framework of Klatt et al., (2015) [2] and determined by our pilot study that examined the effect of manipulating these parameters on the magnitude of postural sway responses.   - Removing vision - Stimulating vestibular input via head movement in yaw and pitch planes - Reducing somatosensory input by standing on foam or on an uneven surface - Reducing base of support (wide base to semi-tandem to tandem stance to single leg) - Combinations of the above   A typical sample progression of static standing exercises over the course of four weeks is provided in the following example. As can be seen, the patient starts with standing on foam with eyes closed and feet apart, and thus is forced to rely on vestibular cues for balance. This can be progressed in week 2 by reducing the base of support to a feet together position. Next, in week 3, vestibular stimulation can be introduced by moving the head and biomechanical demands raised by standing in tandem. In week 4, the very challenging single leg stance can be performed with eyes closed and head movements, but this is done on a firm surface because of the difficulty. Note that this example is just provided for the purpose of illustration, and that the PT may use any of the strategies listed above to increase the difficulty so that the intensity of the exercises is equal to that assigned for the intervention.     \| Exercise Category \| Level 1 \| Level 2 \| Level 3 \| Level 4 \| \| --- \| --- \| --- \| --- \| --- \| \| Standing Balance \| Eyes closed  Foam  Head still  Feet apart  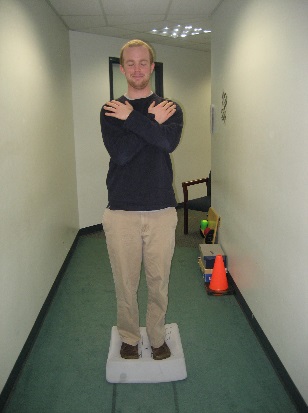 \| Eyes closed  Foam  Head still  Feet together  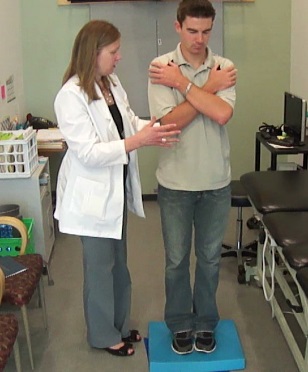 \| Eyes open  Foam  Head turns  Tandem stance  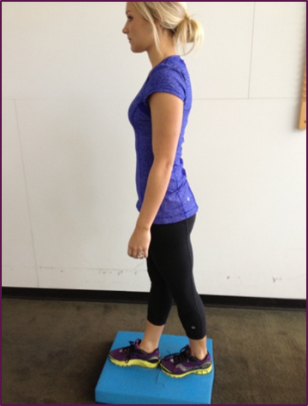 \| Eyes closed  Firm  Head turns  Single Leg Stance  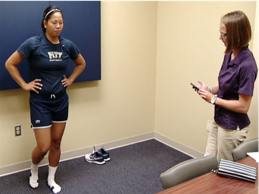 \|   **Dynamic Balance**  The primary goal of dynamic balance exercises is to perturb the base of support and provide greater vestibular stimulation, especially to the otolith organs via head linear acceleration. Examples of exercises include weight shifting, stepping, lunging, and jumping. The exercises can be made more difficult by the following strategies. The exercises are listed in order of increasing difficulty, as recommended in the exercise progression framework of Klatt et al., (2015) [2].   - Removing vision - Stimulating vestibular input via head movement in yaw and pitch planes - Reducing somatosensory input by standing on foam or on an uneven surface - Changing directions - Combinations of the above   A typical sample progression of dynamic balance exercises over the course of four weeks is provided in the following example. As can be seen, the patient starts with marching in place with eyes open, both on level surface and on foam. In week 2, changes in vertical position are performed by squatting and picking up objects. In weeks 3 and 4, lunges are performed in a variety of directions and with head turns to get multi-planar vestibular stimulation. Note that this example is just provided for the purpose of illustration, and that the PT may use any of the strategies listed above to increase the difficulty so that the intensity of the exercises is equal to that assigned for the intervention.   \| Exercise Category \| Week 1 \| Week 2 \| Week 3 \| Week 4 \| \| --- \| --- \| --- \| --- \| --- \| \| Dynamic Balance \| Marching in Place  Level and Foam  30 sec  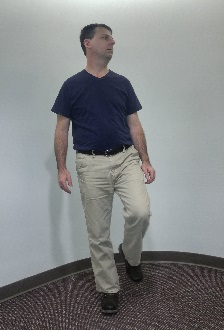 \| Squatting and picking up objects  Feet together  10 times; 2 sets  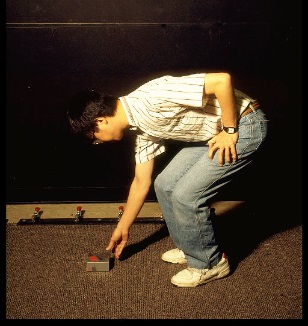 \| Alternating forward lunges  10 times; 2 sets  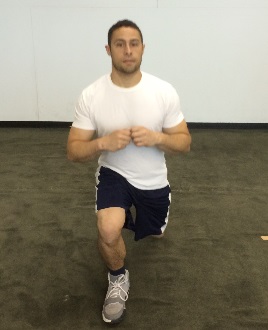 \| Forward lunges with Head/body turns  20 times   \|   **Gait**  The primary goal of gait exercises is to promote functional mobility in a variety of contexts that simulate real-world activities. Gait exercises also promote sensory integration by altering the sensory input and to challenge the postural control system by changing the biomechanical demands. Sensory input is altered by closing eyes, standing on foam or uneven surfaces, or by stimulating the vestibular system with head movement.  The exercises can be made more difficult by the following strategies. The exercises are listed in order of increasing difficulty, as recommended in the exercise progression framework of Klatt et al., (2015) [2].   - Stimulating vestibular input via head movement in yaw and pitch planes - Removing vision - Reducing somatosensory input by walking on foam or on an uneven surface - Reducing base of support (wide base to semi-tandem to tandem stance to single leg) - Changing direction - Performing light aerobic activities - Combinations of the above   A typical sample progression of gait exercises over the course of four weeks is provided in the following example. The patient begins with walking with head turns in a quiet environment. In week 2, forward and backward walking is performed with head turns in a busy gym environment, which is progressed in week 3 by performing with a very narrow base of support. In week 4, the patient moves onto light aerobic jogging to prepare for exertional therapy. Note that this example is just provided for the purpose of illustration, and that the PT may use any of the strategies listed above to increase the difficulty so that the intensity of the exercises is equal to that assigned for the intervention.     \| Exercise Category \| Week 1 \| Week 2 \| Week 3 \| Week 4 \| \| --- \| --- \| --- \| --- \| --- \| \| Gait \| Walking forward  Horizontal and vertical head turns  Quiet environment  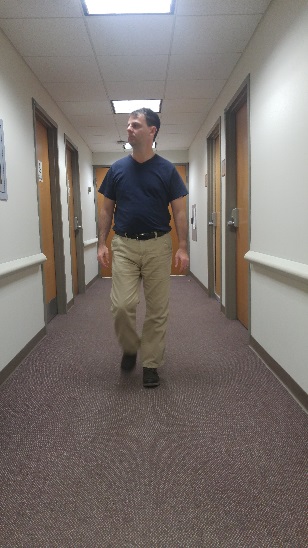 \| Walking forward and backward  Horizontal and vertical head turns  Busy environment  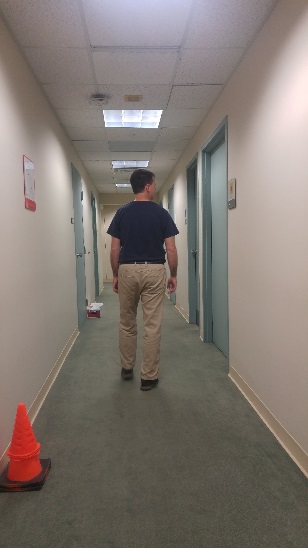 \| Walking forward  Horizontal and vertical head turns  Tandem position  Busy environment  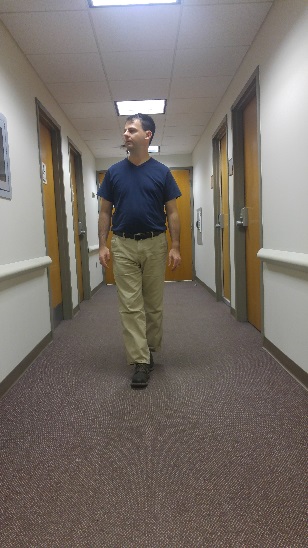 \| Jogging with forward and backward head and body turns  Busy environment  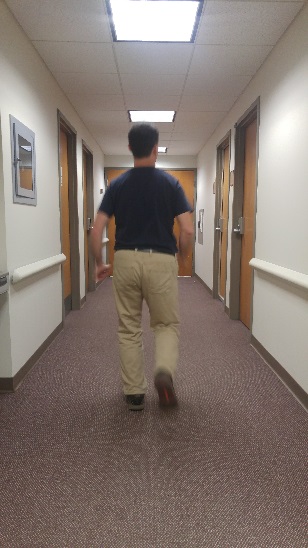 \|   **Compliance**  Compliance with specific vestibular exercises will be collected via the daily text messaging surveys. Daily automated text reminders will be sent each evening to remind and briefly assess compliance with the subject’s assigned intervention. The text response will take subjects to a link with four items regarding:   1. Did you perform your assigned VESTIBULAR concussion intervention today? **YES/NO** 2. How would you rate your overall concussion symptoms today? **BETTER/NO CHANGE/WORSE** 3. How would you rate your percent back to normal from your injury today? **0-100% analog scale** 4. Do you have any problems, questions or concerns that you would like to tell us at this time? **YES/NO** | |
|  |  |  |
|  |  |  |
| Sleep | If participants are adjudicated to have sleep, the intervention will involve:   1. CBT-i Coach App for Sleep Promotion 2. Daytime Recommendations Sleep Monitoring via Fitbit Inspire2 3. Compliance   **CBT-i Coach App for Sleep Promotion**  Each participant will be provided with the free App- CBT-i Coach- which will provide sleep assessments, real-time changes to improve sleep hygiene, and detailed information about their sleep habits via a log. The CBT-i Coach also includes mindfulness-based training for sleep promotion. The app guides participants through the process of learning about sleep, developing positive sleep routines, and improving their sleep environments. It provides a structured program that teaches strategies to improve sleep and help alleviate symptoms of insomnia. <https://mobile.va.gov/app/cbt-i-coach#AppDescription>  Patients will utilize three different components of CBT-i Coach:    1. My sleep -> participants will enter sleep-related information (very similar to PSQI [see Study Measures] questions) once per day.  Various metrics of quality of sleep are calculated and placed on a graph.  After 7 days, the app will provide a prescribed sleep/wake time based on results.  Participants will also fill out the Insomnia Severity Index (ISI) daily.    2. Tools -> Participants will incorporate suggestions into their nighttime routine based on their unique sleep issue and cycle. There are relaxation and mindfulness exercise incorporated into this component of the CBT-i Coach.    3. Reminders -> The CBT-i Coach provided daily reminders to complete sleep entries, the ISI survey and other components of the intervention.  **Daytime Recommendations**  In addition to the app and behavioral sleep regulation components, participants may be instructed to perform their assigned physical activity (e.g., 30 min of walking at brisk pace) in the morning prior to work, school, etc. Participants will be instructed to follow a set sleep-wake schedule to promote appropriate length (6-9 hours) and quality of sleep; and hygiene including sleep restrictions (i.e., avoiding prolonged [>30 min] napping and sleeping other than at night); and not using technology, eating, drinking alcohol, or exercising within 2 hours of bedtime.  **Sleep Monitoring**  Sleep will be tracked via the FitBit Inspire 2; when the participant’s data is downloaded the study team will have access to sleep data and how many hours participants got each day during their study participation.  **Compliance**  Compliance with specific sleep interventions will be collected via the daily text messaging surveys. Daily automated text reminders will be sent each evening to remind and briefly assess compliance with the subject’s assigned intervention. The text response will take subjects to a link with four items regarding:   1. Did you perform your assigned SLEEP INTERVENTION today? **YES/NO** 2. How would you rate your overall concussion symptoms today? **BETTER/NO CHANGE/WORSE** 3. How would you rate your percent back to normal from your injury today? **0-100% analog scale** 4. Do you have any problems, questions or concerns that you would like to tell us at this time? **YES/NO**   ***Note About Additional Sleep Issues:***  If participants report or are diagnosed with more substantial sleep issues (e.g., sleep apnea, sleep disordered breathing) will be referred for a comprehensive sleep medicine history and physical examination will be performed and interpreted by a licensed neurologist specializing in sleep medicine. These patients may receive additional interventions including positive pressure therapy, oral appliance therapy, or behavioral management therapies per standard of care. | |
|  |  |  |
| Autonomic | If participants are adjudicated to have autonomic dysregulation, the intervention will involve:   1. Graded aerobic exertion 2. Activity Monitoring 3. Compliance   **Graded Aerobic Exertion**  A graded aerobic exercise protocol derived from a recent successful RCT demonstrating reduced time to clearance in an aerobic exercise group compared to a stretching group with acute mTBI (Leddy et al., 2019). Participants will be instructed to reach a target goal HR, operationally defined as 80% of HR at symptom exacerbation on the BCTT based on their first visit evaluation. Target heart rates are determined bi-weekly as long as the participant is symptomatic, by completing another BCBT every two weeks under clinical supervision. The graded exertion intervention will take approximately 20 min to complete each day. Participants can walk or jog if they do not have access to cycle or treadmill equipment. Participants will be instructed to stop their home exercise session if their symptoms increase by 2 or more points from their pre-exercise symptom level (on a 10-point visual analog scale[VAS]) or at 20 minutes, whichever came first. They will be told to rest apart from the prescribed exercise and not participate in physical activity beyond the interventions including but not limited to gym class, sport practice, etc.  The table below shows an example of a graded exertion program. The program assigned to participants will be tailored to their needs and current symptom reporting and abilities.   \| **Stage of Rehabilitation** \| **Exercise**  **Intensity** \| **Physical Therapy Program:**  **Vestibular Dysfunction** \| \| --- \| --- \| --- \| \| **STAGE 1**  **Recommendations:** exercise in quiet area, no impact activities; balance and vestibular treatment by specialist (prn); limit head movement/position change; limit concentration activities \| **BORG RPE:**  Target 11-13 \| - Stationary aerobic conditioning: walking or bike - Static balance activities - Exercises that limit head movements (weight machines, squats/lunges with focusing) - Core exercises without head movements \| \| **STAGE 2**  **Recommendations:** exercise in gym areas recommended; use various exercise equipment; allow some positional changes and head movement; low level concentration activities (counting repetitions) \| **BORG RPE:**  Target 13-15 \| - More progressive dynamic aerobic conditioning: elliptical, treadmill walking, progress to treadmill jogging - Balance activities with head movements - Resistance exercises with head movements (example: lateral squats with head movement) - Low intensity sport specific activities - Core exercises with head movements (ex: side planks with arm /head turn, bicycles, Russian twists) \| \| **STAGE 3**  **Recommendations**: any environment ok for exercise (indoor, outdoor); integrate strength, conditioning, and balance/proprioceptive exercise; can incorporate concentration challenges (counting exercises, visual games) \| **BORG RPE:**  Target 13-15 \| - Moderately aggressive aerobic exercise (intervals, pyramids, stair running) - All forms of strength exercises - Dynamic warm-ups - Impact activities (running, plyometrics) - Challenge positional changes (burpees, mountain climbers) - More aggressive sport-specific activities (80% max hr) \| \| **STAGE 4**  **Recommendations:** continue to avoid contact activity, but resume aggressive training in all environments \| **BORG RPE:**  Target 15+ \| - 80% max exertion sport-specific activities avoiding contact \| \| **STAGE 5**  **Recommendations:** Initiate contact activities as appropriate to sport activity; full exertion activities for sport activities \| **BORG RPE:**  Target 15+ \| - Full physical training activities with contact \|   **Activity Monitoring**  Each participant will be provided with a FitBit Inspire 2, and data will physical activity be downloaded and analyzed, thus allowing the study team to review, monitor and adjust levels of physical activity for participants, as well as their HR while performing the graded exertion sessions.  **Compliance**  Compliance with specific exertional/autonomic exercises will be collected via the daily text messaging surveys. Daily automated text reminders will be sent each evening to remind and briefly assess compliance with the subject’s assigned intervention’s frequency and intensity. The text response will take subjects to a link with four items regarding:   1. Did you perform your assigned EXERTION-AUTONOMIC concussion intervention today? **YES/NO** 2. How would you rate your overall concussion symptoms today? **BETTER/NO CHANGE/WORSE** 3. How would you rate your percent back to normal from your injury today? **0-100% analog scale** 4. Do you have any problems, questions or concerns that you would like to tell us at this time? **YES/NO** | |
